# Developing a high-throughput screening method for threonine overproduction based on an artificial promoter

**DOI:** 10.1186/s12934-015-0311-8

**Published:** 2015-08-22

**Authors:** Ya’nan Liu, Qinggang Li, Ping Zheng, Zhidan Zhang, Yongfei Liu, Cunmin Sun, Guoqiang Cao, Wenjuan Zhou, Xiaowei Wang, Dawei Zhang, Tongcun Zhang, Jibin Sun, Yanhe Ma

**Affiliations:** College of Biotechnology, Tianjin University of Science and Technology, Tianjin, 300222 People’s Republic of China; Key Laboratory of Systems Microbial Biotechnology, Chinese Academy of Sciences, Tianjin, 300308 People’s Republic of China; Tianjin Institute of Industrial Biotechnology, Chinese Academy of Sciences, Tianjin, 300308 People’s Republic of China; School of Pharmaceutical Sciences, Nanjing Tech University, Nanjing, 211800 People’s Republic of China

**Keywords:** High-throughput screening, Threonine biosensor, Threonine production, *Escherichia coli*

## Abstract

**Background:**

l-Threonine is an important amino acid for animal feed. Though the industrial fermentation technology of threonine achieved a very high level, there is still significant room to further improve the industrial strains. The biosensor-based high-throughput screening (HTS) technology has demonstrated its powerful applications. Unfortunately, for most of valuable fine chemicals such as threonine, a HTS system has not been established mainly due to the absence of a suitable biosensor. In this study, we developed a HTS method to gain high-yielding threonine-producing strains.

**Results:**

Novel threonine sensing promoters including cysJp and cysHp were discovered by proteomic analyses of *Escherichia coli* in response to extracellular threonine challenges. The HTS method was constructed using a device composed of the fused cysJp and cysHp as a promoter and a linked enhanced green fluorescent protein gene as a reporter. More than 400 strains were selected with fluorescence activated cell sorting technology from a library of 20 million mutants and tested within 1 week. Thirty-four mutants have higher productivities than the starting industrial producer. One mutant produced 17.95 % more threonine in a 5-L jar fermenter.

**Conclusions:**

This method should play a functional role for continuous improvement of threonine industry. Additionally, the threonine sensor construction using promoters obtained by proteomics analyses is so convenient that it would be easily extended to develop HTS models for other biochemicals.

## Background

Threonine is the third bulky amino acid in animal feed industry. Its global annual output reached 300 thousand metric tons in 2014. It was predicted that the annual growth rate of threonine market will exceed 20 % in the next 5–10 years. The market volume will reach 500–600 thousand tons in 3 years (data from a commercial report by askci.com). The large market attracted many powerful companies. Meihua, C.J., Ajinomoto, ADM, Evonik are among the key worldwide players. Though the industrial fermentation technology of threonine achieved a very high level, generally greater than a concentration of 120 g/L and a yield of 0.53 g threonine/g glucose corresponding to the 0.62 g threonine/g glucose of theoretical yield, there is still significant room to further improve the process.

Threonine industrial strains were generated by two major approaches in general, “rational metabolic engineering” and “random mutagenesis and screening”. In the rational metabolic engineering approach, the synthesis of the target product is enhanced by genetic manipulation of the relevant genes according to the existing knowledge or new information from systems biology analyses. The said relevant genes can be the genes directly participating in the biosynthesis pathways, or indirectly affecting the biosynthesis such as transcriptional regulators, membrane transporters, degradation pathways [1–3]. Lee and his colleagues demonstrated how to use this approach to design a good threonine producer from scratch. They achieved a threonine concentration of 82.4 g/L and a yield of 0.393 g threonine/g glucose [4]. With the accumulation of knowledge on cellular and metabolic regulation at both mechanism and kinetics levels, rational design and subsequent metabolic engineering represents a more and more important tool for creating industrial producers. However, most of the strains used in the industrial practice for bulky chemical production were not developed by means of pure rational metabolic engineering mainly due to the poor understanding of the cells. The non-rational approach, mutagenesis and screening is still playing an essential role for bulky chemical production including threonine.

The random mutagenesis and screening approach typically involves the screening of improved strains with targeted phenotypes from a large mutant library. The mutant library is often generated by physical (e.g. UV light, ARTP: atmospheric and room temperature plasma mutagenesis system) [5], chemical (e.g. nitrosoguanidine) or biological (e.g. dnaQ mutants) [6] mutagenesis factors. The generation of the library can be a random process while the screening is preferred to be highly targeted at clear phenotypes [7]. However, the targeted chemicals often do not confer an easy-to-detect phenotype to the better-producing cells. In spite of high flexibility and accuracy, the conventional evaluation methods such as chromatography and mass spectrometry are too time-consuming and laborious to handle millions of mutants. For this reason, smart technologies including molecular sensors have been developed to allow pre-selection of better producers from large population of mutants. Well-designed sensors can specifically translate invisible product concentrations into detectable signals such as fluorescence output which can then be easily handled by the fluorescence-activated cell sorting (FACS) device [8, 9].

In nature, the cells evolved diverse molecular devices such as transcriptional factors, allosteric proteins, enzymes and riboswitches to sense the intracellular or extracellular chemicals. Recently, genetically encoded biosensors have been developed on the basis of such devices, including sensors for amino acids and their precursors such as l-lysine, l-arginine, l-serine, *O*-acetyl-l-serine [10], *O*-acetyl homoserine [11], l-methionine, l-leucine, l-isoleucine, l-valine [12, 13], and a sensor for oxygen [14]. Several sensors have been successfully used in HTS to improve the strain productivity [10, 15], to deregulate the allosteric inhibition of enzymes by cellular metabolites [16], and to increase enzymatic activities [17]. Modern HTS technology becomes a new and powerful tool for both biological discovery and rational or semi-rational design inspired by reverse engineering [10, 18, 19].

The biosensor-based HTS technology has demonstrated its powerful applications. Unfortunately, for most of valuable fine chemicals such as threonine, the HTS system is not established mainly due to the absence of a suitable biosensor. In this study, we would like to identify suitable biological devices to construct an artificial l-threonine sensor for screening high-productive strains for l-threonine.

## Results

### Selection of a potential promoter capable of responding to threonine

**Fig. 1:**
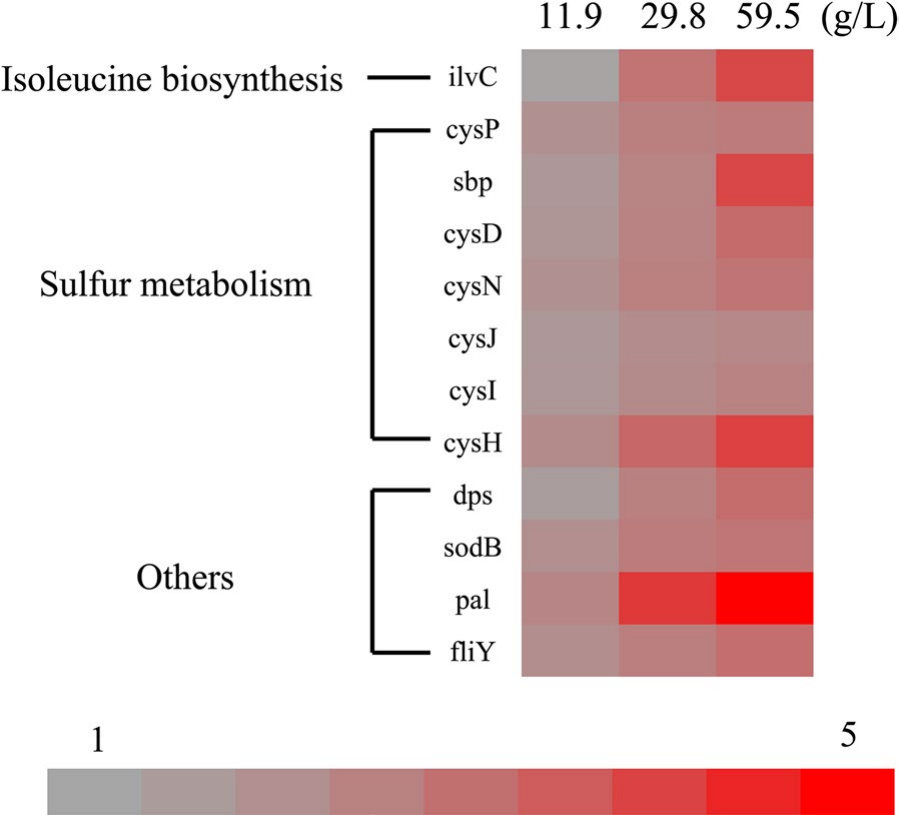
Hierarchical clustering of expression levels of selected genes. iTRAQ-labelled proteomic analyses were carried out using *E. coli* MG1655 cells treated with 0, 11.9, 29.8, 59.5 g/L threonine added in the cultures, respectively. The *color bars* represent the fold change of the expression of selected genes in the treated group versus that of the untreated group (0 g/L threonine). The functional hierarchy was applied according to the information in Ecocyc (www.ecocyc.com).

We carried out iTRAQ-labelled proteomic analyses of *E. coli* MG1655 cells treated with 0, 11.9, 29.8, 59.5 g/L threonine added in the cultures, respectively, and 1,632 proteins were detected, representing approximately 40–45 % of the predicted proteins in *E. coli*. More than 400 proteins showed enhanced expression in response to the increase of threonine concentrations in comparison to the untreated group, wherein 27 proteins have upregulation of more than 1.5 fold in both samples treated with 29.8 and 59.5 g/L threonine. When a cutoff of p value less than 0.05 was applied, 12 proteins were selected (Fig. 1), including proteins of the sulfate metabolism branch of the cysteine biosynthesis pathway encoded by the genes *cysD*, *cysN*, *cysJ*, *cysI*, *cysH*, components of sulfate transporters CysP and Sbp, *ilvC*-encoding acetohydroxy acid isomeroreductase in l-isoleucine biosynthesis pathway from threonine, and additional proteins related with stress response such as those encoded by genes *sodB*, *dps*, *pal* and *fliY*. According to these results and some preliminary tests, we decided to examine the possibility of merging the promoter of the operon *cysJ*-*cysI*-*cysH* [20] and the promoter of *cysH* [21] to build an artificial fusion promoter cysJHp to gain better response to threonine.

### The fusion cysJHp promoter near linearly responded to extracellular threonine

**Fig. 2:**
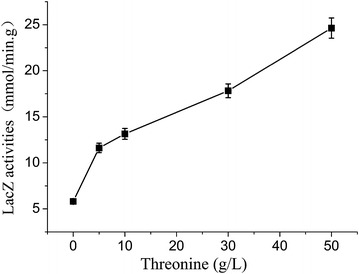
The specific activities of *lacZ* in MGl655(pTZL1) exposed to threonine added to the cultures. Data are the mean and standard deviation of independent triplicates.

To further quantify the regulatory role of l-threonine on the control of cysJHp expression, a plasmid pTZL1 carrying the promoter cysJHp and the reporter gene *lacZ* was constructed and used to transform MG1655. The expression levels of the reporter gene *lacZ* in MGl655(pTZL1) were tested against the addition of different levels of threonine in LB medium. As shown in Fig. 2, the specific activities of *lac*Z under the control of cysJHp ranging from 5.81 ± 0.26 U/mg to 24.64 ± 1.10 U/mg, showing an almost linear ascending trend as the concentrations of threonine supplementation increase from 0 to 50 g/L, clearly suggesting the fusion promoter cysJHp is induced by extracellular threonine. To exclude the influence possibly exerted by osmotic pressure from threonine, NaCl in a concentration of 30 g/L, instead of threonine, was added into the culture. The specific activity of lacZ in this case was determined as low as 4.82 ± 0.21 U/mg, close to the basal expression level without threonine addition, indicating that the osmatic pressure did not significantly affect the induction of cysJHp in the test condition. The close-to-linear response of the cysJHp promoter to the external addition of threonine hints that it would be an ideal candidate to sense the production capacity of the cell.

### The cysJHp responded to intracellular threonine

To test if endogenous threonine or the production capacity plays a similar regulatory role on the control of cysJHp expression, the LacZ activities were examined in a threonine-producing strain ThrH(pTZL1) and a strain ThrL(pTZL1), the latter used as a control, as well as MG1655(pTZL1). The strains were cultured in shaking flasks containing the fermentation medium for 34 h. The specific activity of LacZ in ThrH(pTZL1) was almost twice as high as those of ThrL(pTZL1) and MG1655(pTZL1) (Table 1). The intracellular and extracellular concentrations of threonine in the threonine producing strain ThrH(pTZL1) were higher than those of the two non-producing strains. The production capacity is positively correlated to intracellular/extracellular concentrations of final product threonine as we proposed above. The intracellular concentration is also consistent to the induction strength of the fusion promoter cysJHp expressed as the activity of LacZ. The results gave hints that the cysJHp promoter is able to sense the intracellular threonine concentration and is a good indicator of the threonine production capacity.

**Table 1:** Comparison of LacZ expression under the control of cysJHp in strains with different threonine-producing capacities

Strains	ThrH(pTZL1)	ThrL(pTZL1)	MG1655(pTZL1)
Specific activity of LacZ (U/mg)	97.30 ± 6.53	50.44 ± 0.64	47.71 ± 3.21
Intracellular threonine concentration (g/L)^a^	3.19 ± 0.17	0.10 ± 0.02	0.10 ± 0.01
Extracellular threonine concentration (g/L)^b^	5.83 ± 0.02	Not-detected	Not-detected

^a^Determined by using the LC–MS/MS method.

^b^Determined by using the high-pressure liquid chromatography (HPLC) method. Data are the mean and standard deviation of independent triplicates.

### Establishment of a biosensor from the cysJH promoter to work with FACS

**Fig. 3:**
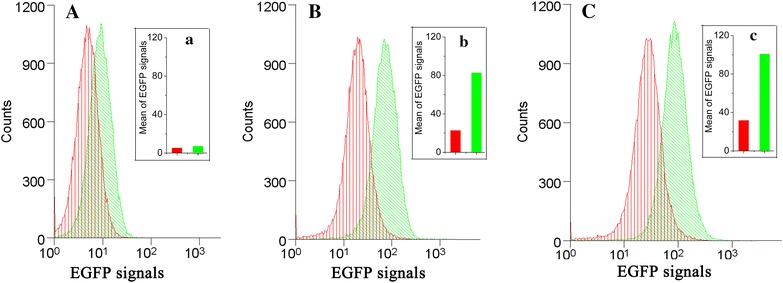
The fluorescence signals of ThrH(pTZL2) and ThrL(pTZL2) cells at different fermentation times. **A** and *a* 0 h; **B** and *b* 10 h. **C** and *c* 24 h. The fluorescence signals of ThrL(pTZL2) were shown in *red*, while those of ThrH(pTZL2) were shown in *green*.

LacZ activity delivered quantitative information to the promoter activity. But it was not convenient to work with FACS for fast cell-based screening. To establish a biosensor capable of working with FACS, a plasmid of pTZL2 carrying an *egfp* gene under the control of the *cysJH* promoter was constructed and used to transform the ThrH and ThrL strains. The two recombinant strains were cultivated separately in shaking flasks with fermentation medium. Samples were taken at 0, 10 and 24 h and submitted to FACS. The result is shown in Fig. 3. The two strains showed no clear difference in fluorescence at 0 h (Fig. 3A, a). However, as the fermentation process went on, the cells exhibited increasing fluorescence signals (Fig. 3a–c), and the difference of the two strains became larger with increasing fermentation time (Fig. 3A–C). From 10-h on, the two strains can be clearly distinguished as two groups. The results indicated that the *egfp* under the control of the promoter cysJHp is able to function as a sensor to work with the FACS system and be used for HTS.

### High-throughput screening a mutant library by FACS facilitated with the threonine sensor

**Fig. 4:**
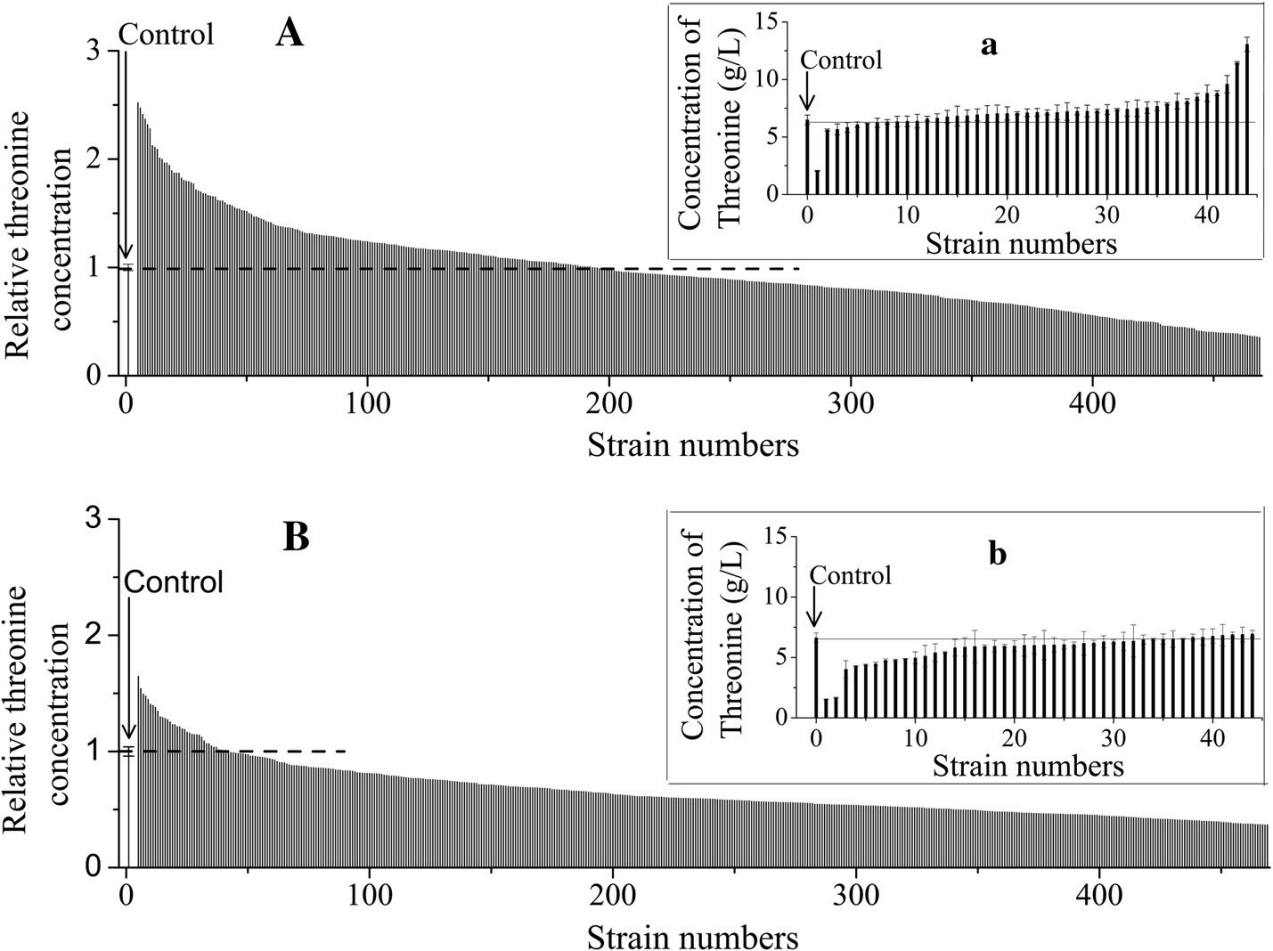
The threonine production by the FACS-selected and randomly-selected mutants. Strain ThrH(pTZL2) was used as the control. The threonine production of FACS-selected and randomly-selected mutants was firstly detected by the rough but fast ninhydrin spectrophotometer method as shown as **A**, **B**, respectively. The threonine concentration of the control culture was set as 1. The Top 44 FACS-selected and randomly-selected mutants were further cultured and measured by HPLC shown in *a* and *b*, respectively. Data in *a* and *b* are the mean and standard deviation of independent triplicates.

The modified industrial producer ThrH(pTZL2) was treated with the ARTP mutation system as described [22] to build a mutant library. The treated cell suspension was cultivated in a shaking flask with the fermentation medium for 12 h to allow accumulation of intracellular threonine and induction of the EGFP protein. Using FACS, 465 cells were selected from about 2 × 10^7^ mutants in the library (named as FACS-selected) and tested in fermentation medium with 96-well plates. Three un-treated ThrH(pTZL2) colonies were inoculated into independent wells in each block as controls. In addition, cells with a gate of 100 % were also selected (named as randomly-selected) and cultured for comparison. Threonine concentrations were determined by the ninhydrin coloration method. The mutants with highest production of threonine were further confirmed by HPLC analysis. As a result, more than 40 % of FACS-selected cells produced higher amount of threonine than the control strain (Fig. 4A), while that was only 10 % for the randomly-selected cells (Fig. 4B). The top 44 threonine hyper-productive mutants in the two groups (Fig. 4A, B) were further cultured in 96-well plates, and threonine concentrations were analyzed using the more accurate HPLC method (Fig. 4a, b). For the FACS-selected strains, 34 mutants produced more threonine than the original strain, and 29 and 19 mutants produced more than 5 % and 10 % more threonine, respectively (Fig. 4a), whereas none of the randomly-selected mutants produced more than 5 % more threonine than the parent strain (Fig. 4b). These results confirmed that FACS with the aid of the threonine sensor elevated the positive rate of the screening.

### Fermentation test of the selected mutants

The threonine production capacities of several selected mutants were further tested in a 5-L bioreactor. As shown in Table 2, in comparison to the parent strain ThrH(pTZL2), the best mutant ThrH-27(pTZL2) produced more threonine with less glucose consumption after 47 h of fermentation. The production yield increased from about 0.39 to 0.46 (g threonine/g glucose), with relative improvement of 17.95 %.

**Table 2:** Comparison of threonine production of the selected mutants with the parent strain

Strains	Threonine concentration (g/L)	Total threonine in a batch (g)	Yield (threonine/glucose, g/g)
ThrH(pTZL2)	117.60 ± 2.17	322.84 ± 5.61	0.39 ± 0.01
ThrH-27(pTZL2)	123.61 ± 3.07	349.12 ± 1.87	0.46 ± 0.00
ThrH-5(pTZL2)	127.92 ± 0.73	323.98 ± 7.83	0.42 ± 0.01
ThrH-33(pTZL2)	113.45 ± 1.31	325.89 ± 3.68	0.42 ± 0.00

Data are the mean and standard deviation of independent triplicates.

## Discussion

Industrial strain is one of the most important factors for successful industrial production of a target chemical. Rational design together with modern metabolic engineering of industrial strain has been demonstrated to be a valuable method but with most trials in vain. With the aid of modern HTS technique, traditional random mutagenesis becomes efficient to obtain an improved hyper-producing strain and can even synergize with other rational or semi-rational design approaches to offer good mutants for evaluation of the hypothesis or build new recombinants by reverse engineering [10, 18, 19]. The key for HTS is a rapid and effective screening model. As most biochemical synthesis processes occur within the cells, an ideal screening system should make use of the extensive cellular biosensors which are involved in the nature to sense and respond to diverse endogenous and environmental chemical signals. Molecular biochemistry studies have disclosed many macromolecules as biosensors. Binder et al. [10] demonstrated the use of the transcriptional factor LysG-based biosensor to construct a HTS device for screening lysine-producing mutants from a wild type model strain *C. glutamicum* ATCC 13032. As a result, many better mutants were obtained. Interestingly, sequence analysis to the mutants found new gene mutations contributing to lysine hyper-production. In another report [19], the leader and sensor region of the *E. coli lysC* gene was used to construct an artificial lysine sensor. Using this sensor, the expression level of PPC was successfully optimized for lysine production with an *E. coli* strain. Due to the absence of a suitable biosensor for most of valuable fine chemicals such as threonine, developing methods to discover potential biosensors becomes very important. In this study, we applied the proteomic tool to analyze the response of a lab strain *E. coli* MG1655 upon exposure to extracellular threonine administration. A novel synthetic threonine biosensor cysJHp was built according to the proteomic findings and successfully applied to establish a HTS protocol for screening mutants for elevated threonine production. It took about only 1 week to complete the established screening procedure for a 2 × 10^7^-mutant library, which is 10^4^–10^5^ times faster than the traditional mutagenesis and flask culture-based screening methods.

Suitable biosensors are important for successful screening. Ideal biosensors should have a number of good features: sensitivity, dynamic range, and specificity. As sensitivity the sensor should response strongly to the environmental changes so that the response could be detected easily by a detector. As dynamic range, the sensor should give a linear response to a large range of signal. As specificity, the sensor should respond only to the desired objective. In this study, iTRAQ-labeled LC–MS/MS-based proteomic analyses gave relative quantification information on cellular proteins in response to four different extracellular threonine challenges (0, 11.9, 29.8, 59.5 g/L). CysJ, CysI and CysH are among the strongly induced proteins, and the expression levels increased linearly as the increase of l-threonine concentrations in the culture medium. In *E. coli*, these three genes are organized as one operon with two promoters, cysJp locating at the upstream of *cysJ* [20] and cysHp locating at the upstream of *cysH* [21], generating two transcripts of *cysJIH* and *cysH*, respectively. In *E. coli*, cysJp was reported to be controlled by the LysR-type transcriptional activator CysB and by the inducer *N*-acetyl-l-serine [23]. The regulatory mechanism of cysHp is still not clear. According to the proteomic data, the expression levels of CysJ and CysI were similar but that of CysH was significantly higher than the other two. It is reasonable to speculate that both of cysJp and cysHp could be affected by threonine independently. To enhance the strength of output signals, the promoters cysJp and cysHp were merged together to form a synthetic promoter cysJHp. The binary promoter cysJHp may not directly respond to threonine in nature. In our further experiments, we confirmed that the artificial promoter responded to the extracellular change of a large range of threonine strongly and near linearly. Although the non-specificity of the promoter may lead to selection of mutants producing chemicals other than threonine, the next experimental verification by the ninhydrin reaction method and HPLC can give accurate results. We believe that it is sufficient to use the synthetic promoter cysJHp to develop a HTS screening system to screen mutants with improved threonine-producing capability starting from an existing threonine producer at this stage.

Proteomic study would easily give many candidates which all respond to the targeted challenge. It is very important to select good potential promoters for building artificial biosensors from the upregulated gene sets. As many other biological products, threonine is firstly synthesized within the cell and then transported outside of the cell membrane. Optimization of the synthesis efficiency should cause the increase of its intracellular concentration. This is why a molecular sensor sensing the intracellular concentration of a biochemical can be used to evaluate the production capabilities of the mutants in most cases. One exception is improved membrane transportation systems, which may also lead to higher production efficiency but lower intracellular concentration. The intracellular biosensor-based approach is not suitable to screen mutations with improved transportation efficiency. It is not known whether the upregulation of transporter proteins CysP and Sbp found in our threonine challenge study is related to the intracellular threonine or not. No matter yes or no, the promoters for transporter proteins should not be the priority for selection. SodB is a stress response protein. Its overexpression might be the response to osmotic pressure caused by higher concentrations of threonine salts but not threonine itself. Therefore it was also not tested further.

Although the laboratory strain *E. coli* MG1655 was used to test the proteomic response to extracellular threonine in this study, it is more preferable to use industrial strain to conduct the test if the objective is clearly set to improve the production of this strain. Different strains, even those with close evolutionary relation in phylogenetic analysis, may harbor different but important genetic variations and regulations. In our study, we found that the promoter of gene *ilvC* showed good response to extracellular threonine addition in MG1655 but had no response in strain ThrL (data not shown).

Similarly, we need to assure that the sensor really responds to endogenous chemical signal instead of extracellular administration. In our study, the strain ThrH is an industrial threonine producer. ThrH(pTZL1) produced 5.83 ± 0.02 g/L threonine in the uncontrolled fermentation condition with flasks. ThrL is a control strain of ThrH. Both ThrL and MG1655 are non-threonine producing strains. The intracellular threonine concentration of ThrH(pTZL1) was 3.19 ± 0.17 g/L, more than 30 times higher than those of ThrL(pTZL1) and MG1655(pTZL1) (about 0.1 g/L). When LacZ enzyme was used as a reporter to work with the synthetic cysJHp promoter, LacZ activity of ThrH(pTZL1) was almost two times as high as those of ThrL(pTZL1) and MG1655(pTZL1).

The detection method is crucial for an efficient HTS method. It must be fast, simple, and able to be realized with high-throughput instruments. In this study, threonine concentrations were firstly estimated by reading the OD_570_ developed from the threonine-ninhydrin reaction. The color development reaction and OD_570_ measurement could be conveniently carried out with a 96-well thermal cycler and a microplate reader. The process is fast and cheap, the throughput is thousands times faster than chromatography and mass spectrometry methods. Although factors such as other amino acids in the crude cell cultures may interfere with the analytical results, it is not important in this study since threonine is the major product of the threonine high-producing starting strain. In the next procedure, HPLC measurement was applied and confirmed that the fast but non-selective ninhydrin-based approach was actually efficient and accurate enough to pick out positive mutants.

## Conclusions

By combining proteomic study we built a synthetic promoter cysJHp which strongly and near linearly responded to threonine. We built a biological senor making use of this promoter together with a signal gene *egfp* and at the first time to the best of our knowledge constructed a HTS model for screening threonine over-producing mutants successfully. Using this method, we obtained 44 strains with higher productivities than the original strain from 2 × 10^7^ mutant cells within 1 week. One mutant produced 17.95 % more threonine than the original strain in a 5-L jar fermenter. This method should play a functional role for continuous improvement of threonine production industry.

It should be mentioned that the mechanism how the binary promoter cysJHp responds to threonine remains unclear. But the situation did not prevent cysJHp from becoming a useful sensor to develop a HTS screening system. Since it is very convenient to use transcriptomes or proteomics to discover the overexpression patterns in response to extracellular challenges, we expect that this method would be easily extended to develop high throughput screening models for other biochemicals.

## Materials

l-threonine and ninhydrin were supplied by Sinopharm Chemical Reagent Co., Ltd (Tianjin, China). *O*-nitrophenyl-β-d-galactopyranoside (ONPG) and *O*-nitrophenyl were supplied by solarbio (Beijing, China). Dimethyl sulphoxide (DMSO) was supplied by Fine Chemical Institute (Tianjin, China). Citric acid, sodium citrate and 3-morpholinopropanesulfoinc acid (MOPS) were supplied by Amresco (USA). All other chemicals used were of analytical grade. Restriction endonucleases were purchased from Fermentas (USA). DNA polymerases were obtained from Transgene (Beijing, China). The T4 DNA ligase was purchased from New England Biolabs, Inc. (Beijing, China).

### Strains and plasmids

The strains, plasmids and primers used in this study are listed in Table 3. Other strains were constructed based on them.

**Table 3:** The strains, plasmids and primers used in this study

	Characteristics	Source
Strains		
MG1655	A substrain of *E. coli* strain K-12, with a relevant GenBank accession No: NC_000913.3	Lab stock
ThrH	A threonine-producing *E. coli* mutant containing a plasmid-borne threonine biosynthesis operon *thrABC* with streptomycin resistance as selective marker	Lab stock
ThrL	Derived from the ThrH strain by deleting the plasmid	Lab stock
Plasmids		
pET21a-egfp	An enhanced green fluorescent gene (*egfp*) linked to the plasmid pET21a	Lab stock
pSB4K5-I52002	Km^r^, GenBank accession NO: EU496099	[33]
pTZL1	Km^r^, constructed by cloning the cysJHp promoter and the *lacZ* gene into pSB4K5-I52002	This study
pTZL2	Km^r^, constructed by cloning the cysJHp promoter and the *egfp* gene into pSB4K5-I52002	This study
Primers		
CysJp-1	CGCCCTAGGATCCGTTGCGCAAAATCGCTGATTTATC	This study
CysJp-2	CAAGCGACGCCAGGATTTCCGGTAAGCAAAGCTGTTTCTG	This study
CysHp-1	CAGAAACAGCTTTGCTTACCGGAAATCCTGGCGTCGCTTG	This study
CysHp-2	TTACCGCGCGGTGCCTTGCCTGATGCGAC	This study
lacZP-1	TATGGCGCGCCTTTAAGAAGGAGATATACATATGACCATGATTACGGATTC	This study
lacZP-2	GCCACTAGTTTATTTTTGACACCAGACCAACTGGTAATGGTAGCG	This study
EgfpP-1	TATGGCGCGCCTTTAAGAAGGAGATATACATATGGTGAGCAAGGGCGAGGAGC	This study
EgfpP-2	GCCACTAGTTTACTTGTACAGCTCGTCCATGCCGAGAGTGATCCCGG	This study
B4K5P-1	GCGTACCTAGGGAATTCGAGTCACTAAGGGCTAACTAAC	This study
B4K5P-2	GCCACTAGTAGCGGCCGCTGCAGGAGTCAC	This study

For plasmids and strains construction, the target nucleotides were obtained and cloned into corresponding plasmids which were then used to transform target strains for subsequent experiments. The promoter cysJp of the operon *cysJIH* [24–26] was cloned by PCR using the genome of *E. coli* MG1655 as a template with a pair of primers CysJP-1 and CysJP-2. The promoter cysHp of the *cysH* gene [21] was cloned by a similar method using a pair of primers CysHP-1 and CysHP-2. Then, fusion PCR was carried out with the primers CysJP-1 and CysHP-2 using the PCR products containing cysJp and cysHp as templates. The fusion PCR product was named cysJHp with *Avr*II and *Asc*I restriction sites at each end. The *lacZ* gene was amplified from the *E. coli* MG1655 genomic DNA with a pair of primers lacZP-1 and lacZP-2. The *egfp* gene was amplified from the plasmid pET21a-egfp with a pair of primers EgfpP-1 and EgfpP-2. Both of the PCR products of *lacZ* gene and *egfp* gene had *Asc*I and *Spe*I restriction sites at each end. A fragment of plasmid pSB4K5-I52002 was amplified with a pair of primers B4K5P-1 and B4K5P-2 with *Avr*II and *Spe*I restriction sites, respectively. The PCR products including cysJHp promoter, *lacZ* gene, and plasmid pSB4K5-I52002 fragment were digested with the corresponding restriction enzymes and ligated together to form a plasmid pTZL1. A plasmid pTZL2 was constructed similarly with the reporter gene *egfp* instead of *lacZ*. Transformation of the plasmids into different *E. coli* host strains generated MGl655(pTZL1), ThrH(pTZL1), ThrL(pTZL1), ThrH(pTZL2) and ThrL(pTZL2).

### Medias and cultivation conditions

Cells were routinely cultured with the Luria–Bertani (LB) medium. Cells for proteomic analyses were cultivated with the M9 minimal medium containing 2 g/L yeast extract. For cultivation and fermentation evaluation of the mutants, fermentation medium containing (g/L) 50 glucose, 10 (NH_3_)_2_SO_4_, 2 KH_2_PO_4_, 4 yeast extract powder, 1 MgSO_4_·7H_2_O was used. MOPS was supplemented at a final concentration of 0.4 mol/L to the fermentation medium to buffer the pH when shaking flasks or 96-well microtiter plates were used. According to the resistance of strains, kanamycin was added at a final concentration of 25 mg/L and/or streptomycin was added at a final concentration of 50 mg/L.

Agar plates were incubated at 37 °C for 24 h. All cultivations with shaking flasks were done at 37 °C, 220 r/min with 20 mL medium in 500 mL shaking flasks. For the fermentation test in flasks, 1 % volume of overnight LB culture was used as the seed. Fermentation in 96-well microtiter plates was carried out as following: colonies from agar plates were inoculated in 96-well Deep Well Assay Blocks (Corning Costar 3960, square V-bottom, 2 mL) containing 300 μL of the fermentation culture in each well, and then incubated at 37 °C, 850 r/min for 24 h in a Microtron shaker (Infors). Fermentation in a 5-L jar fermenter (Shanghai BaoXing Bio-engineering Equipment Co., Ltd, China) was carried out as following: seeds were prepared as two successive pre-cultures, the first and second with the LB and the fermentation medium, respectively. After the second pre-culture grew to an OD_600_ about 5, 100 mL of the second seed culture was moved to the 5-L jar fermenter containing 1,900 mL of fermentation medium. Fed-batch fermentation was carried out for 47 h at 37 °C, pH 7.0, and dissolved oxygen 20 % or higher. Glucose solution of 800 g/L was continuously supplied to control glucose concentration at 5–10 g/L in the culture.

### The proteomic analyses of MG1655 in response to threonine addition

*E. coli* MG1655 was cultivated to the exponential phase, then threonine was added into the cultures to various concentrations (0, 11.9, 29.8, 59.5 g/L, respectively). Cells were cultivated for another 2 h, harvested by centrifugation at 12,000 r/min for 10 min at 4 °C, and resuspended in a lysis buffer (Tris–HCl pH 7.6 at 100 mmol/L,DTT 100 mmol/L, cocktail slice 10 mL: obtained from Calbiotech, Inc). The cells were sonicated on ice, centrifuged at 12,000 r/min for 10 min at 4 °C. The supernatants were transferred into new tubes and the concentrations of proteins were quantitated using a 2D-Quant Kit (purchased from GE healthcare). The protein samples were then treated by Isobaric tags for relative and absolute quantitation (iTRAQ) using a method modified from a former report [27]. Proteins from each sample (100 µg) were reduced, cysteine blocked, digested and labeled with respective isobaric tags using an iTRAQ Reagent 4-plex Kit (Applied Biosystems, Foster City, CA, USA) according to the manufacturer’s protocol. All of the labelled samples were pooled, mixed equally, and fractionated by a Nexera UHPLC LC-30A system (Shimadzu, Japan) at a flow rate of 0.8 mL/min using a Durashell C18 column (5 μm, 150 Å, 4.6 mm × 250 mm, Agela Technologies) resistant to high pH values. The HPLC gradient consisted of buffer A (0.1 % formic acid, 2 % acetonitrile) and buffer B (0.1 % formic acid, 95 % acetonitrile) with buffer B varying from 5 to 40 %. The collected fractions were combined into 10 samples and concentrated to dryness for later analyses.

A NanoLC system (NanoLC-2D Ultra, Eksigent) equipped with a triple TOF 5600 mass spectrometer (AB SCIEX, USA) was used for the liquid chromatography-quadrupole mass spectrometry (LC–MS/MS) analysis. Peptides were trapped on a NanoLC trap column (Chromxp C18CL, 3 μm, 120 Å, 350 μm × 0.5 mm, Eksigent) and then eluted onto an analytical column (Chromxp C18CL, 3 μm, 120 Å, 75 μm × 150 mm, Eksigent) and separated by a 120-min gradient buffer A and B with B from 5 to 35 % (buffer A: 2 % acetonitrile; buffer B: 98 % acetonitrile, 0.1 % formic acid) at a flow rate of 300 nL/min. Full-scan MS was performed in positive ion mode with a nano-ion spray voltage of 2.5 kv from 350 to 1,500 (*m*/*z*), with up to 30 precursors selected for MS/MS (*m*/*z* 100–1,500) if exceeding a threshold of 125 counts per second (counts/s). Peptides with +2 to +5 charge states were selected for MS/MS. The collision energy (CE) for collision-induced dissociation was automatically controlled using an information-dependent acquisition CE parameter script to achieve the optimum fragmentation efficiency.

Data analyses were performed using a method modified from a former report [27]. The MS data acquisition was performed with the Analyst v1.6 software (AB SCIEX, USA). Protein identification and quantification was performed using the ProteinPilot v4.5 software (AB SCIEX, USA). The parameters were set as follows: (1) sample type, iTRAQ 8-plex (peptide labeled); (2) cysteine alkylation, methyl methanethiosulfonate; (3) digestion, trypsin; (4) instrument, triple TOF 5600; (5) ID focus, biological modifications; and (6) search effort, thorough ID. In the iTRAQ quantitation, the peptide for quantification was automatically selected by the Pro Group™ algorithm to calculate the reporter peak area. More than two peptides and a strict unused confidence score >1.3 were used as the qualification criteria, which corresponds to a peptide confidence level of 95 %. The resulting data set was auto bias-corrected to get rid of any variations imparted due to the unequal mixing during combining different labeled samples.

### Cell mutagenesis

Cells cultured overnight in LB medium were harvested, washed and resuspended in 10 % glycerol with an OD_600_ of 1.0. The cell suspension was treated with an ARTP mutagenesis system for 25 s following a protocol previously reported [22]. The cells treated were cultured in fermentation medium for 12 h and sorted by a FACS system (Beckman Coulter MoFlo XDP).

### Cell analysis and sortation by FACS

Cells were harvested, washed and resuspended in potassium phosphate buffer (PB) (100 mmol/L, pH 7.0) to an OD_600_ of 1.0. Then, EGFP in each cell was analyzed by FACS with the following parameters: excitation at 488 nm, detection fluorescence at 529 ± 14 nm, sample pressure of 60 psi. The nozzle diameter was 70 μm. Sterile filtered phosphate-buffered saline was used as the sheath fluid. Data were analyzed using the Beckman Summit software v5.2. The selection gate was set as 0.01 % of the total cells based on the pre-analysis of the mutant library. The selected cells were pooled in a test tube and then spread on agar plates for overnight cultivation. The colonies were spotted into 96-well microtiter plates containing fermentation medium for the fermentation test.

### Measurement of LacZ specific activities

Cells were harvested, washed and resuspended in PB to an OD_600_ of 3.0. Then, the cells were lysed by sonication and centrifuged at 12,000 r/min for 10 min. The supernatants were retained and protein concentrations were determined using the BCA Protein Assay Kit (Thermo). The assay of LacZ activities was carried out according to a former report [28] in a reaction system containing 114.23 μL of PB, 1.67 μL of MgCl_2_ solution (610 μL of H_2_O, 290 μL of β-mercaptoethanol, 100 μL of MgCl_2_ at 1 mol/L), 74.1 μL of ONPG solution (4 g/L in H_2_O). The formation of ο-nitrophenol was measured at 420 nm with a SpectraMax 190 microplate reader (Molecular Devices, LLC.; Davis 1965). A standard curve of *o*-nitrophenol was also measured. All of the measurements were repeated three times at 37 °C. The specific activities of LacZ were calculated according to the corresponding protein concentrations and *o*-nitrophenol formation. Formation of 1 µmol of *o*-nitrophenol in 1 min by 1 mg total cellular proteins means 1 U/mg.

### Extracellular glucose and threonine analyses

Extracellular glucose was detected using a biosensing analyzer (SBA-40D, Shandong, China). Extracellular threonine was analyzed with two methods. For preliminary detection, a modified spectrophotometric method was used [29]. Supernatants of fermentation broths were diluted ten times. Thirty-seven microliter of each of the dilutions was added to 113 µL of ninhydrin solution (200 mM of citric acid-sodium citrate buffer at pH 6.0 containing ninhydrin and cupric sulfate at 3 and 5 g/L, respectively). After reaction at 95 °C for 15 min in a 96-well thermal cycler (ABI Veriti), 75 μL DMSO was added, and the OD_570_ was detected using a microplate reader (SpectraMax 190, Molecular Devices). For more accurately quantitative analyses, HPLC was applied using a HPLC 1260 Infinity system (Agilent 1260) equipped with a Zorbax Eclipse AAA column (4.6 × 150 mm, 5 µm) and a UV detector. A gradient of 40 mM Na_2_HPO_4_ buffer at pH 7.8 with a gradient solution containing acetonitrile/methanol/water (45:45:10, v/v/v) was used as the eluent. Threonine was detected as its *o*-phthalaldehyde derivative at 338 nm following the post-column derivation method. The concentrations of produced threonine in supernatants were calculated through a calibration curve obtained with the standard solution of threonine added to the assay mixture.

### Measurement of intracellular threonine by LC–MS/MS

Cells were cultured in shaking flasks at 37 °C for 34 h using the fermentation medium, and then separated by a silicone oil centrifugation method [30]. The weight of intracellular threonine was analyzed and quantified by LC–MS/MS. The equipment consists of an Agilent 1260 HPLC system with a Merck column (HILIC, 3.0 μm; 2.1 mm × 100 mm) and a micrOTOF-Q II mass spectrometer (Bruker Daltonik, Germany). The metabolites were separated under a binary gradient elution with a flow rate of 0.2 mL/min at 30 °C. Solvent A was 10 mM ammonium acetate and 0.02 % acetic acid in water-acetonitrile mixture (water:acetonitrile at 10:90). Solvent B was 10 mM ammonium acetate and 0.02 % acetic acid in acetonitrile. The gradient condition in term of percentage of solvent B was raring from 100 to 45 %. The micrOTOF-Q II mass spectrometer was operated in a negative electrospray ionization (ESI) mode with a scan range from 30 to 800 *m*/*z*. The source parameters were set as follows: capillary at −4.2 kV; nebulizer pressure at 1.0 bar; dry gas flow at 6 L/min; dry gas temperature at 180 °C. A Hystar chromatography software (Bruker Daltonik, Germany) was used to control the system and the data were analyzed with the Bruker Compass DataAnalysis software v4.0 (Bruker Daltonik, Germany). The weight of threonine in each of the samples was calculated through the calibration curve obtained with the standard solution of threonine. The corresponding cell aqueous volume was calculated according to the former reports [31, 32]. The intracellular concentration of threonine was calculated accordingly.

## Abbreviations

HTS: high-throughput screening
FACS: fluorescence-activated cell sorting
HPLC: high-pressure liquid chromatography
LC–MS/MS: liquid chromatography–quadrupole mass spectrometry
